# Synthetic GPR datasets to evaluate hybridization inverse approach for pavement tack coat characterization—Geometrical and physical parametric study

**DOI:** 10.1016/j.dib.2025.111794

**Published:** 2025-06-18

**Authors:** Grégory Andreoli, Amine Ihamouten, Xavier Dérobert

**Affiliations:** a Gustave Eiffel University, MAST/MIT – Nantes, F-44344 Bouguenais, France; b Gustave Eiffel University, MAST/LAMES – Nantes, F-44344 Bouguenais, France; c Gustave Eiffel University, GeoEND – Nantes, F-44344 Bouguenais, France; d FI-NDT– Nantes, F-44344 Bouguenais, France

**Keywords:** Pavement interface, Electromagnetism, Machine learning, FWI

## Abstract

The visible surface degradation of pavements is often the result of underlying subsurface defects. The quality of the bond between the wearing course and the binder course is a key factor in limiting issues such as delamination, stripping, etc. The use of non-destructive testing (NDT) methods, such as electromagnetic wave propagation combined with a hybrid data processing approach—machine learning and Full-Waveform Inversion—has recently demonstrated its effectiveness [1]. To support this research, a database was created using the open-source software gprMax [2] for various central frequencies on different two-layered pavement structures, modeled in accordance with current French standards. Variations in geometrical and physical parameters were applied to the wearing course, tack coat, and binder course. The objective is to build a representative database for numerical validation of an innovative hybridization method (machine learning method combined to full-wave form inversion), facilitating the characterization of the tack coat located between the two layers that form the surface layer. The challenge is to discriminate the echo from a thin bituminous subsurface layer using a ground-coupled GPR system (near-field), despite the presence of constructive interference.

Specifications Table

**Table:** 

Subject	Engineering & Materials science
Specific subject area	Numerical Tack coat Characterization with radar methods.
Type of data	Text (*.in), HDF5 (*.out): gprMAx raw format Table (*.csv), Eletrical field following Z and direction (in V/m) and magnetic field following X direction (A/m).l
Data collection	This database is created using the open-source software gprMax for four various central frequencies on different two-layered pavement structures, modeled in accordance with current French standards. Variations in geometrical and physical parameters were applied to each layer to have many realistic configurations.
Data source location	Synthetic dataset generate with the open source software gprMax v.3.1.5: https://www.gprmax.com/ The software simulate electromagnetic wave propagation using Yee’s algorithm to solve Maxwell’s equations applying Finite Difference Time-Domain (FDTD).
Data accessibility	Repository name: Dataverse: recherche.data.gouv.fr Data identification number: 10.57745/IWJJAT Direct URL to data: 10.57745/IWJJAT
Related research article	•The dataset is relative to the article DOI: 10.1016/j.treng.2025.100313 ·Named: Hybridization of machine learning/GPR Full-Waveform Inversion for pavement tack coat characterization: Numerical validation .

## 1 Value of the Data

• The generated datasets consist of synthetic time-domain signals (A-scans) simulating various configurations in terms of thickness and dielectric permittivity of the wearing and binder course in pavement structures. At the interface, a thin layer, representing the tack coat, is also modeled, with thicknesses on the order of millimeters and varying dielectric properties. All geometric and physical parameters are provided for each A-scan. By using the surface layer's dielectric permittivity as *a priori* input for a Support Vector Machine (SVM) machine learning algorithm, both performance and computation time are improved.
• The generation of representative synthetic data for various bonding conditions on structures with variable geometries is highly time-consuming. These data are essential for building high-performance machine learning models;
• In addition to output files in both raw format and tabular form, open access to input files facilitates understanding their creation and enables modifications to expand the database;
• These synthetic databases, generated using gprMax, are made available to the entire scientific and professional pavement community with a focus on GPR applications. They can be useful for the detection and characterization of tack coat interface located between the wearing course and the binder course in accordance with current French standards.

## 2 Background

The purpose of these datasets is to validate the inverse methods allowing characterization of the tack coat in the subsurface using simulated radar data generated with the Finite-Difference Time-Domain (FDTD) modeling tool, gprMax [2]. By inverting the time-domain signals, the objective is to extract quantitative information about the surveyed materials, particularly in terms of layer thickness and electromagnetic properties in two dimensions. To achieve this, a representative database has been generated, structured and processed, exploring a wide range of scenarios and realistic configurations in accordance with current standards while maintaining control over various parameters and degrees of freedom. Among the key variables, variations in the central frequency (900 MHz, 2.6 GHz, 4.5 GHz, and 6 GHz) have been used, along with changes in the thicknesses and dielectric permittivities of the wearing course, tack coat, and binder course. These variations fall within realistic ranges based on existing standards and the components of the asphalt mixtures used, including aggregate type, binder, and compaction levels [3].

## 3 Data Description

The databases consist of raw A-scans data collected for all the configurations described in the previous section using gprMax FDTD model. The first-level folders are dedicated to each center frequency: 900 MHz, 2.6 GHz, 4.5 GHz, and 6 GHz. The obtained signals can also be filtered by subtracting the wave emitted in air. For this, the Ricker folder contains the wavelets propagated in air for each center frequency. Each subfolder indicating the frequency in MHz at the beginning. The data structure tree is described as follows (example for 2.6 GHz):

• Synthetic_GPR_Database
  • /2600MHz
    • /2600_Database_BBSG_TC_2-3_GB
      • 2600_gprMax_Database_BBSG_TC_2-3_GB.zip
        • *“gprMax input file”*.in
        • *“gprMax output file”*.out
        • *“gprMax format to table conversion”*.csv
      • /2600_Information_BBSG_TC_2-3_GB.csv
      • /2600_Distribution_BBSG_TC_2-3_GB.fig
      • /2600_Distribution_BBSG_TC_2-3_GB.tif
      • /2600_Ascans_BBSG_TC_2-3_GB.fig
      • /2600_Ascans_BBSG_TC_2-3_GB.tif
    • /2600_Database_BBSG_TC_6-7_GB
      • /2600_gprMax_Database_ BBSG_TC_6-7_GB.zip
        • *“gprMax input file”*.in
        • *“gprMax output file”*.out
        • *“gprMax format to table conversion”*.csv
      • /2600_Information_BBSG_TC_6-7_GB.csv
      • /2600_Distribution_BBSG_TC_6-7_GB.fig
      • /2600_Distribution_BBSG_TC_6-7_GB.tif
      • /2600_Ascans_ BBSG_TC_6-7_GB.fig
      • /2600_Ascans_ BBSG_TC_6-7_GB.tif
    • /2600_Database_BBTM_TC_2-3_BBSG
      • /2600_gprMax_Database_ BBTM_TC_2-3_BBSG.zip
        • *“gprMax input file”*.in
        • *“gprMax output file”*.out
        • *“gprMax format to table conversion”*.csv
      • /2600_Information_BBTM_TC_2-3_BBSG.csv
      • /2600_Distribution_BBTM_TC_2-3_BBSG.fig
      • /2600_Distribution_BBTM_TC_2-3_BBSG.tif
      • /2600_Ascans_BBTM_TC_2-3_BBSG.fig
      • /2600_Ascans_BBTM_TC_2-3_BBSG.tif
    • /2600_Database_BBTM_TC_6-7_BBSG
      • /2600_gprMax_Database_BBTM_TC_6-7_BBSG.zip
        • *“gprMax input file”*.in
        • *“gprMax output file”*.out
        • *“gprMax format to table conversion”*.csv
      • /2600_Information_BBTM_TC_6-7_BBSG.csv
      • /2600_Distribution_BBTM_TC_6-7_BBSG.fig
      • /2600_Distribution_BBTM_TC_6-7_BBSG.tif
      • /2600_Ascans_BBTM_TC_6-7_BBSG.fig
      • /2600_Ascans_BBTM_TC_6-7_BBSG.tif
• /Ricker (containing the Ricker wavelet propagated in air for each frequency in the database)
  • *“gprMax input file”*.in
  • *“gprMax output file”*.out
  • *“gprMax format to table conversion”*.csv
  • *900_Ricker*.fig
  • *900_Ricker*.tif
  • *2600_Ricker*.fig
  • *2600_Ricker*.tif
  • *4500_Ricker*.fig
  • *4500_Ricker*.tif
  • *6000_Ricker*.fig
  • *6000_Ricker*.tif

**Fig. 1:**
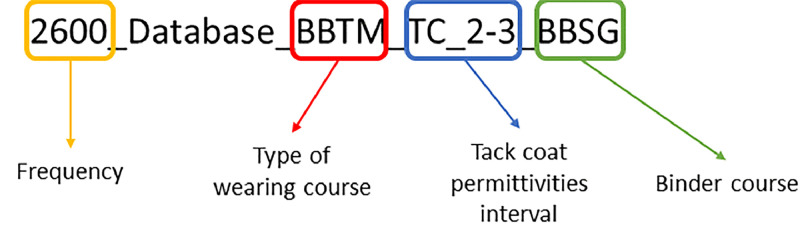
Example folder description.

**Fig. 2:**
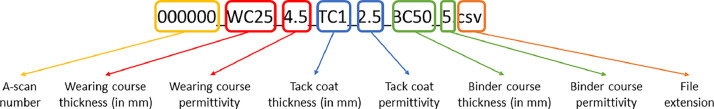
Example file description.
  The *.csv file derived from *.out has three columns: time, electric field following Z direction (E_z_ in V/m) and magnetic field following X direction (H_x_ in A/m).
• A *.csv file beginning by ***XXXX_Information*** (where ***XXXX*** represents the frequency in MHz). This file includes a table with all the information for each A-scan. Each row is structured as follows:
• *.fig and *.tif files beginning by XXXX_Distribution.* show the distribution in thicknesses and permittivities of each layers according to a normal distribution and *.fig and *.tif files beginning by XXXX_Ascans.* show an example A-scans with tack coat thicknesses.

At the second-level, the folders are organized based on the type of modeled two-layered structures according to the nomenclature (Fig. 1):

At the third-level structure, there is:

• A compressed folder containing all the simulated files. For each of them, there is a *.in text file representing the input parameters required for modeling with gprMax, a *.out HDF5 file corresponding to native output data file, and finally a *.csv file, which is the conversion of the *.out file into a directly usable format. Each file follows the naming convention in Fig. 2.

## 4 Experimental Design, Materials and Methods

For all modeled cases (with a *central frequency equal to* 900 MHz, 2.6 GHz, 4.5 GHz, and 6 GHz), a relative conductivity of $\sigma_{r}=\;10^{-5}S/m$, a relative permeability of $\mu_{r}=\;1\;H/m$ and zero magnetic losses were set, corresponding to most geological materials [4]. The modeled two-layered structures are put on a Perfect Electric Conductor (PEC) modeling approach, enabling the identification of the bottom layer. Table 1

**Table 1:** *XXXX_Information* file description.

**Fig. 3:**
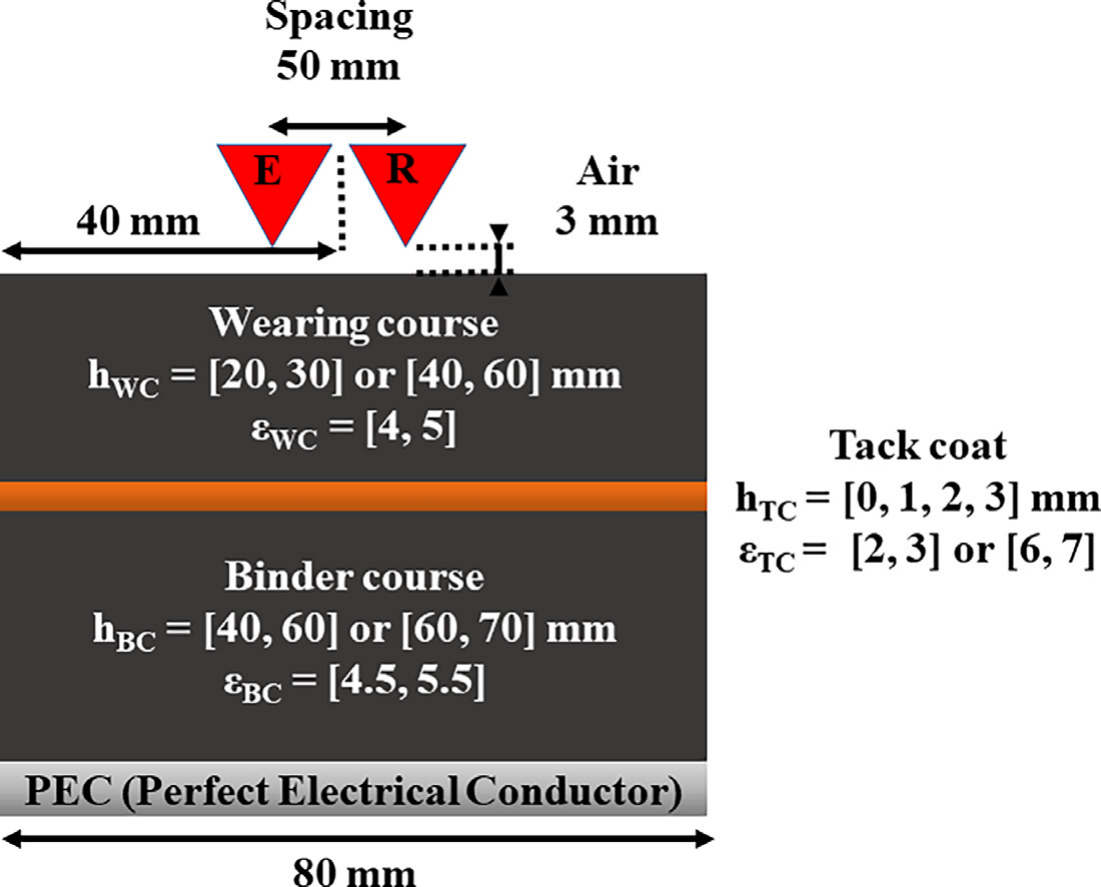
modeled two-layered structures with various geometric and physical parameters.

The sources are modeled as dipole antennas with a transmitter-receiver spacing of 50 mm, propagating a Ricker wavelet toward the two-layered structures with an antenna height of 3 mm above the wearing course (Fig. 3). This antenna height falls within the Rayleigh region (near-field) [1], where constructive interference can prevent signal discrimination, particularly when the wearing course is thin.

The time window (The period during which the receiver antenna captures and records echoes from a transmitted electromagnetic pulse) has been chosen depending on the studied frequency bandwidth and the center frequency fc:

• fc = 900 MHz, Time window = 10 ns, number of points in A-scan = 4241 pts
• fc = 2.6 GHz, Time window = 3 ns, number of points in A-scan = 1273 pts
• fc = 4.5 GHz, Time window = 2.6 ns, number of points in A-scan = 1104 pts
• fc = 6 GHz, Time window = 2.1 ns, number of points in A-scan = 892 pts

*Note: To expand the database, it is possible to vary parameters such as the thickness of each layer, their permittivity and conductivity, as well as the spatial resolution, central frequency, and more.*

The datasets are referenced according to Table 2.

**Table 2:** datasets description.

During the application of the tack coat, it has been observed that under conditions of high humidity, rainfall, or inadequate breaking, water droplets may become trapped at the interface. Given that the permittivity of water is significantly higher (compared to 2.4 at fc=2.6 GHz), the overall permittivity at the interface increases with dispersive effect. To simulate this application anomaly, the authors increased the permittivity range of the tack coat from [2,3] to [6,7]. This approach allowed them to demonstrate the relevance of incorporating dielectric permittivity *a priori* input in a machine learning model, specifically using Support Vector Machine/Support Vector Regression (SVM/SVR) algorithms.

*Note: BBTM is French acronym for Very thin Bituminous Concrete, BBSG is French acronym for Semi-Coarse Bituminous Concrete and GB is French acronym for Bituminous Gravel, h_xx_ is the thickness and ε_xx_ the dielectric permittivity.*

As part of research efforts focused on characterizing thin pavement subsurface layers, the databases generated in this study enabled a feasibility analysis and validation protocol of hybridization approach combining machine learning methods and Full-Waveform Inversion (FWI) [5]. These investigations showed that, by constructing representative databases and processing the data using machine learning techniques (Support Vector Machine), it is possible to classify the bonding state and estimate its thickness [6]. Furthermore, by incorporating an *a priori* physical knowledge (such as dielectric permittivity of the wearing course) into the model as an input parameter, the performance initially provided by the machine learning algorithm are optimized and enhanced [1].

**Fig. 4:**
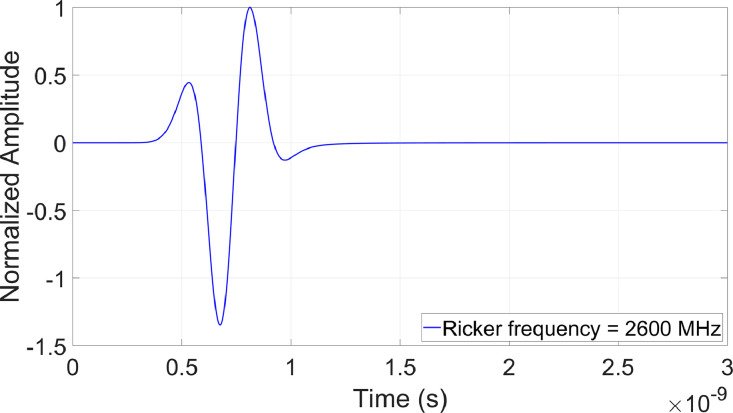
wave propagated in the air at 2.6 GHz.

**Fig. 5:**
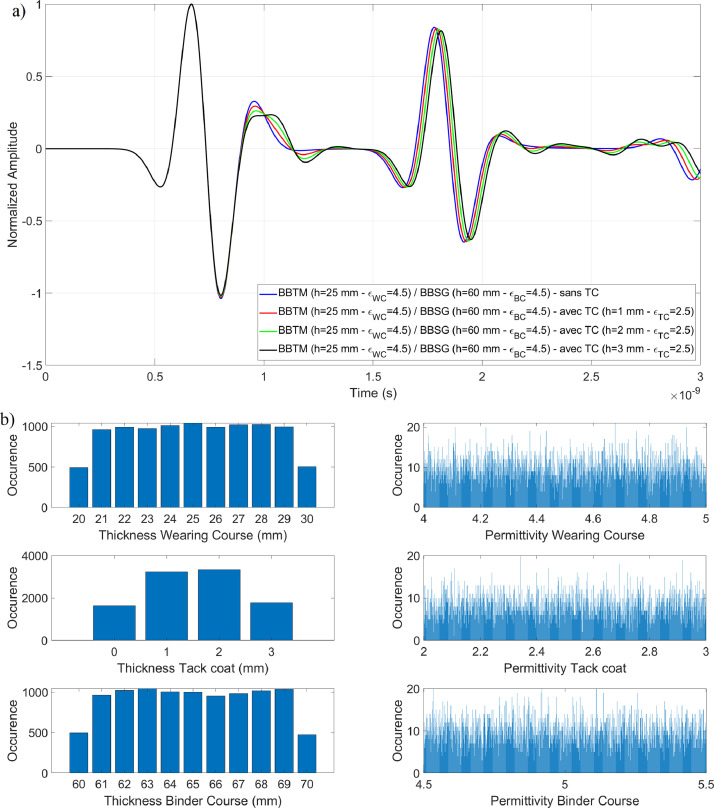
Thicknesses and permittivities of the wearing course, tack coat, and binder course for a BBTM on BBSG structure, with tack coat permittivities $\varepsilon_{WC}\in R^{+}$ varying in the range [2,3] at 2.6 GHz; a) Example of A-scans with various tack coat thicknesses; b) Random distribution of parameters in the training database.
• The testing dataset (Fig. 6). In this example, the dataset consists of BBTM on BBSG with tack coat permittivities $\varepsilon_{WC}\in R^{+}$ varying in the range [2,3] with 4 significant digits at 2.6 GHz.

To this end, two datasets has been considered from which wave in air (Fig. 4) has been subtracted:

• The training dataset (Fig. 5). In this example, the dataset consists of BBTM on BBSG with tack coat permittivities $\varepsilon_{WC}\in R^{+}$ varying in the range [2,3] with 4 significant digits at 2.6 GHz,

**Fig. 6:**
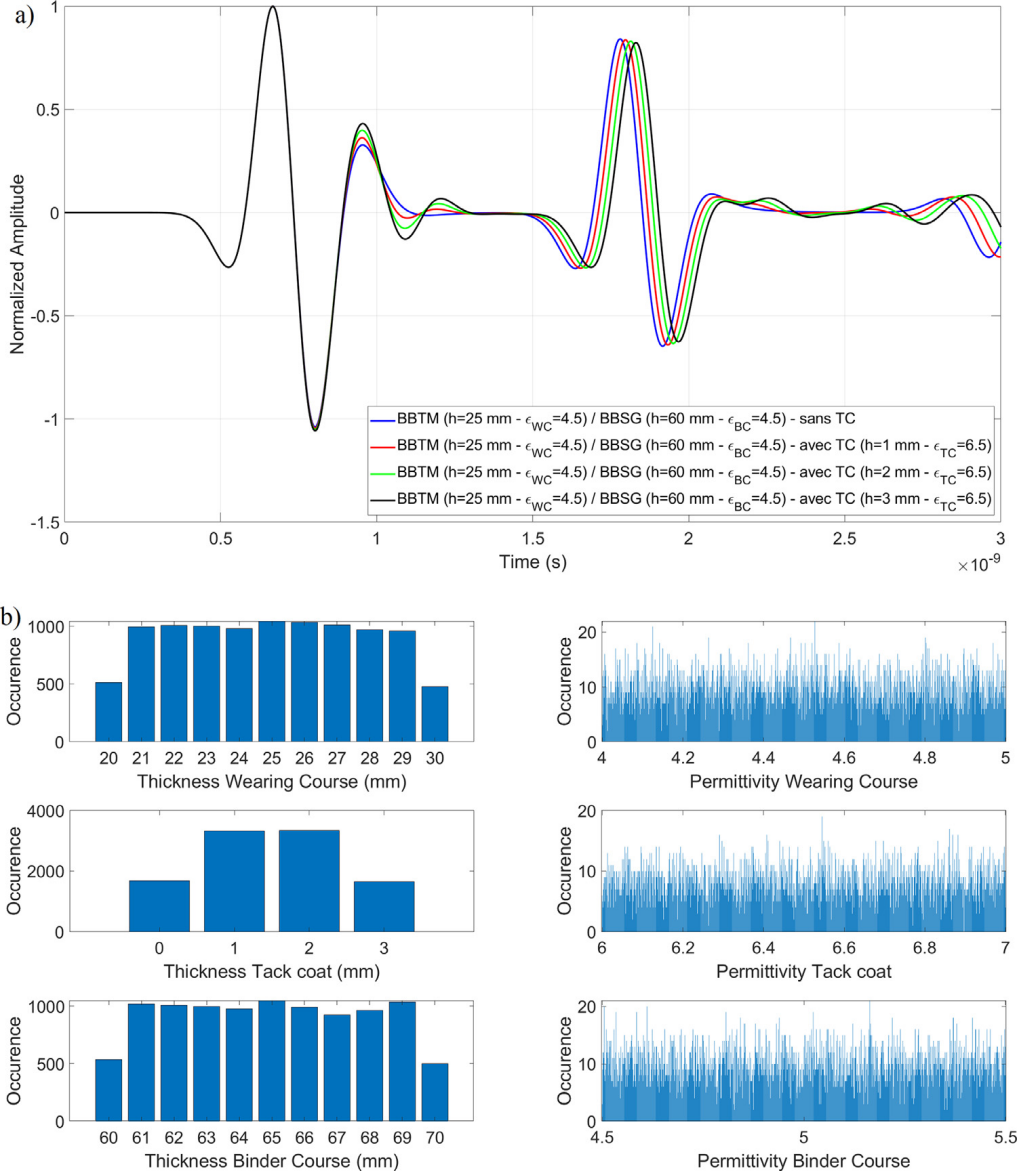
Thicknesses and Permittivities of the wearing course, tack coat, and binder course for a BBTM on BBSG structure, with tack coat permittivities $\varepsilon_{WC}\in R^{+}$ varying in the range [6,7] at 2.6 GHz; a) Example of A-scans with various tack coat thicknesses; b) Random distribution of parameters in the testing database.

**Fig. 7:**
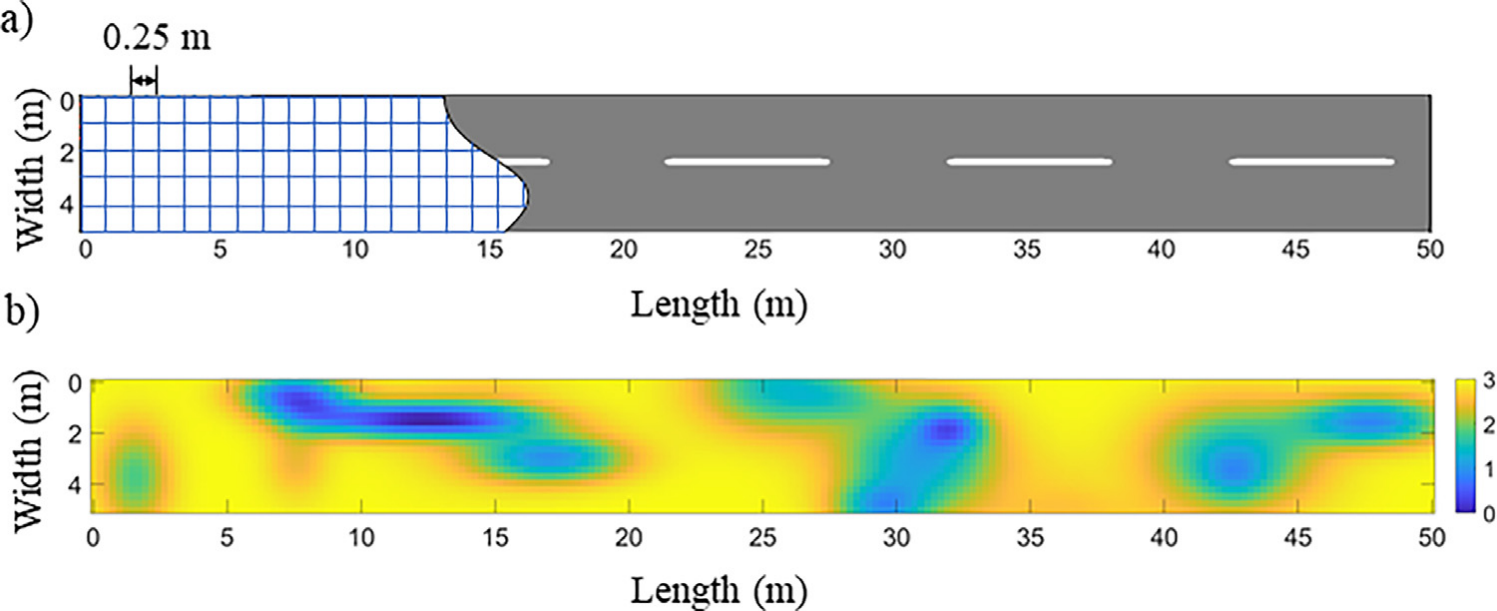
a) Grid of the synthetic pavement structure; b) EM class mapping of the ground truth with various tack coat thicknesses [6].

Thanks to the software Matab (2021a and higher), a bivariate normal distribution is used to modeled an electromagnetic thickness map of the tack coat in two-layered pavement structures, comprising a BBTM (wearing course) over a BBSG (binder course), at a frequency of 2.6 GHz, corresponding to a commercially available GPR system. The mapped section covered 50 meters in length and 5 meters in width, requiring 4,160 A-scans for testing dataset (Fig. 7). To maintain a 70/30 ratio between the training and testing datasets, 9707 A-scans were selected from the 10,000 randomly generated A-scans within the thickness and permittivity ranges defined for each line of the Table 2.

**Fig. 8:**
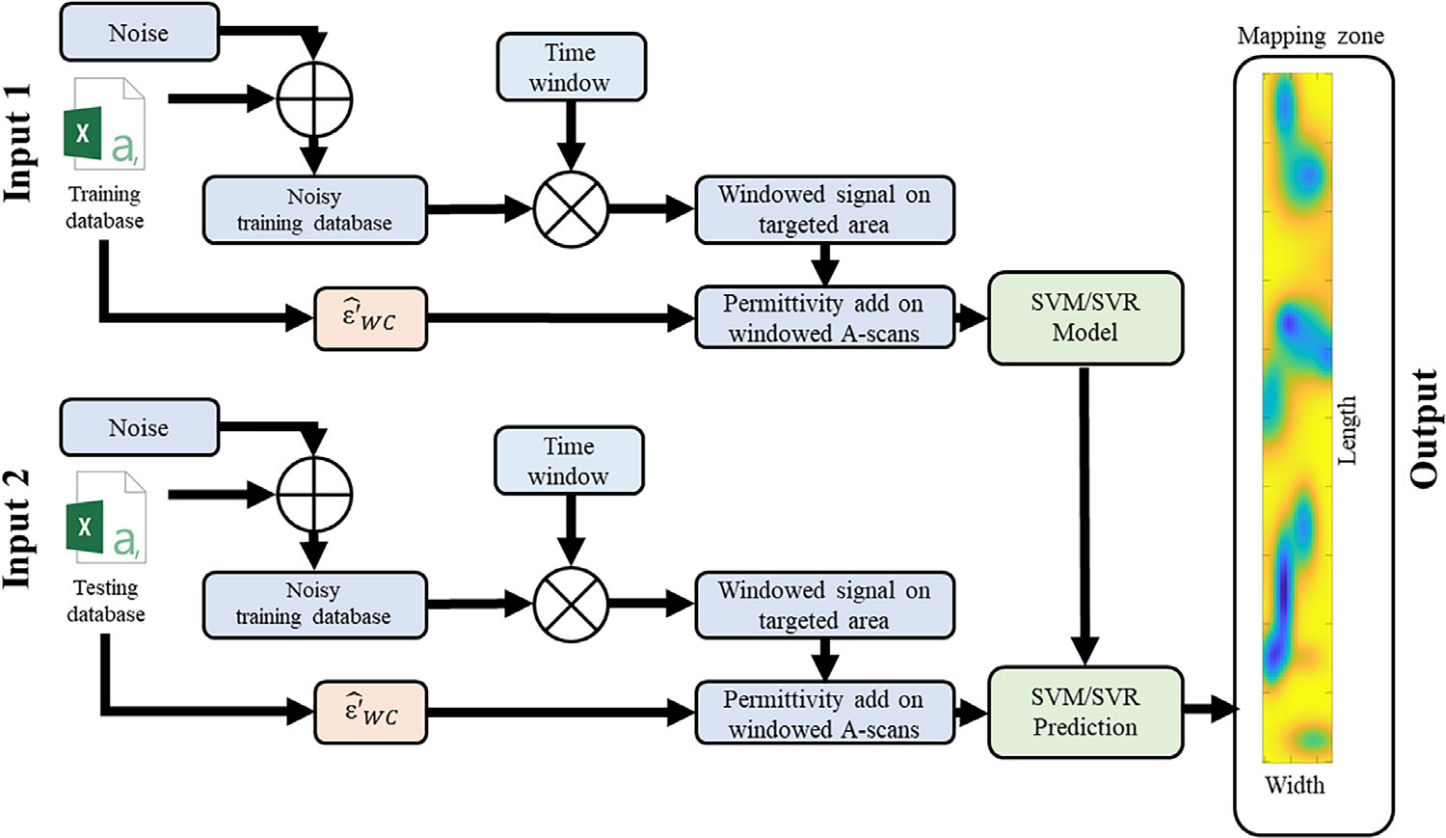
Flowchart illustrating the A–scans processing hybrid method.

To evaluate hybridization method developed in this research, we followed the methodology described in the flowchart below (Fig. 8). The raw dataset, consisting of full A-scans without any preprocessing, is referred to as the **global approach**.

**Fig. 9:**
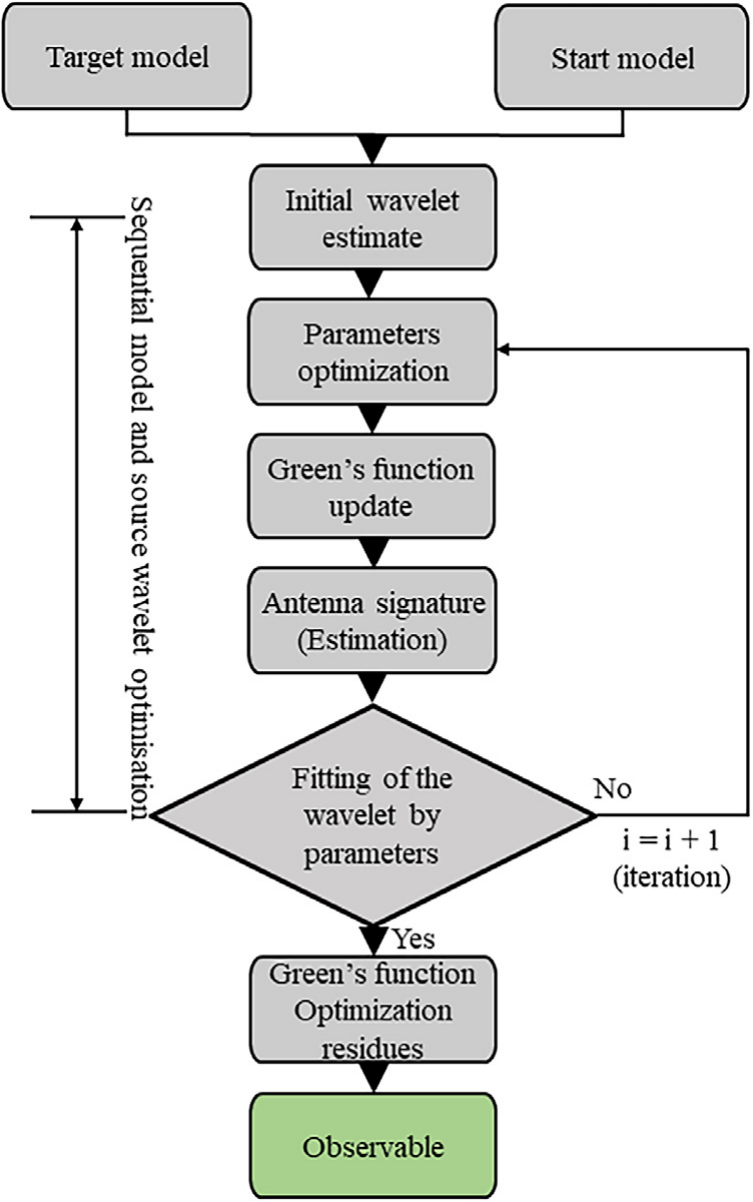
Flowchart describing GPR data processing using FWI to extract the wearing course permittivity for hybridization [1].

**Fig. 10:**
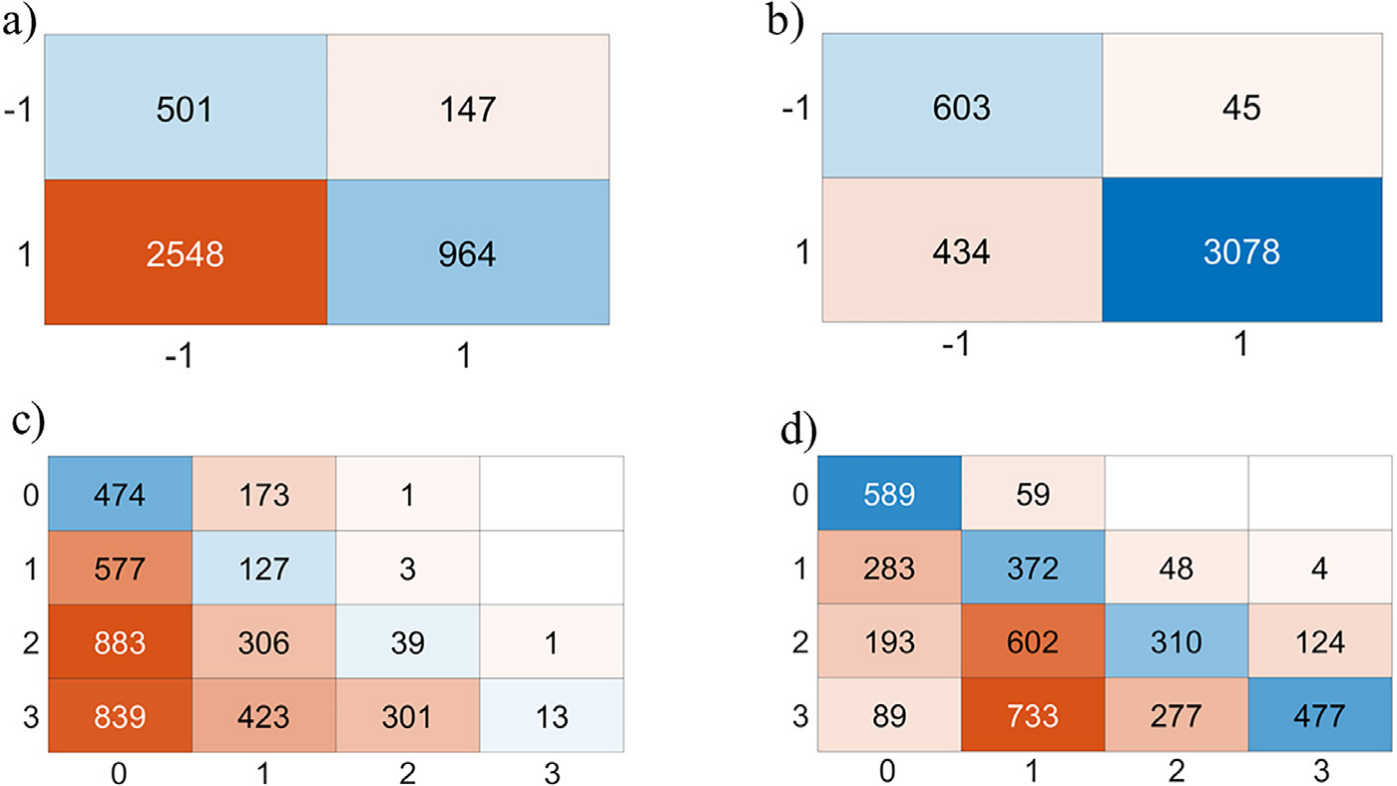
Confusion matrices of the tested database (SNR = 20 dB): a) Two-Class SVM, global approach, without hybridization; b) Two-Class SVM with hybridization; c) Multi-Class SVM, global approach, without hybridization; d) Multi-Class SVM with hybridization.

**Fig. 11:**
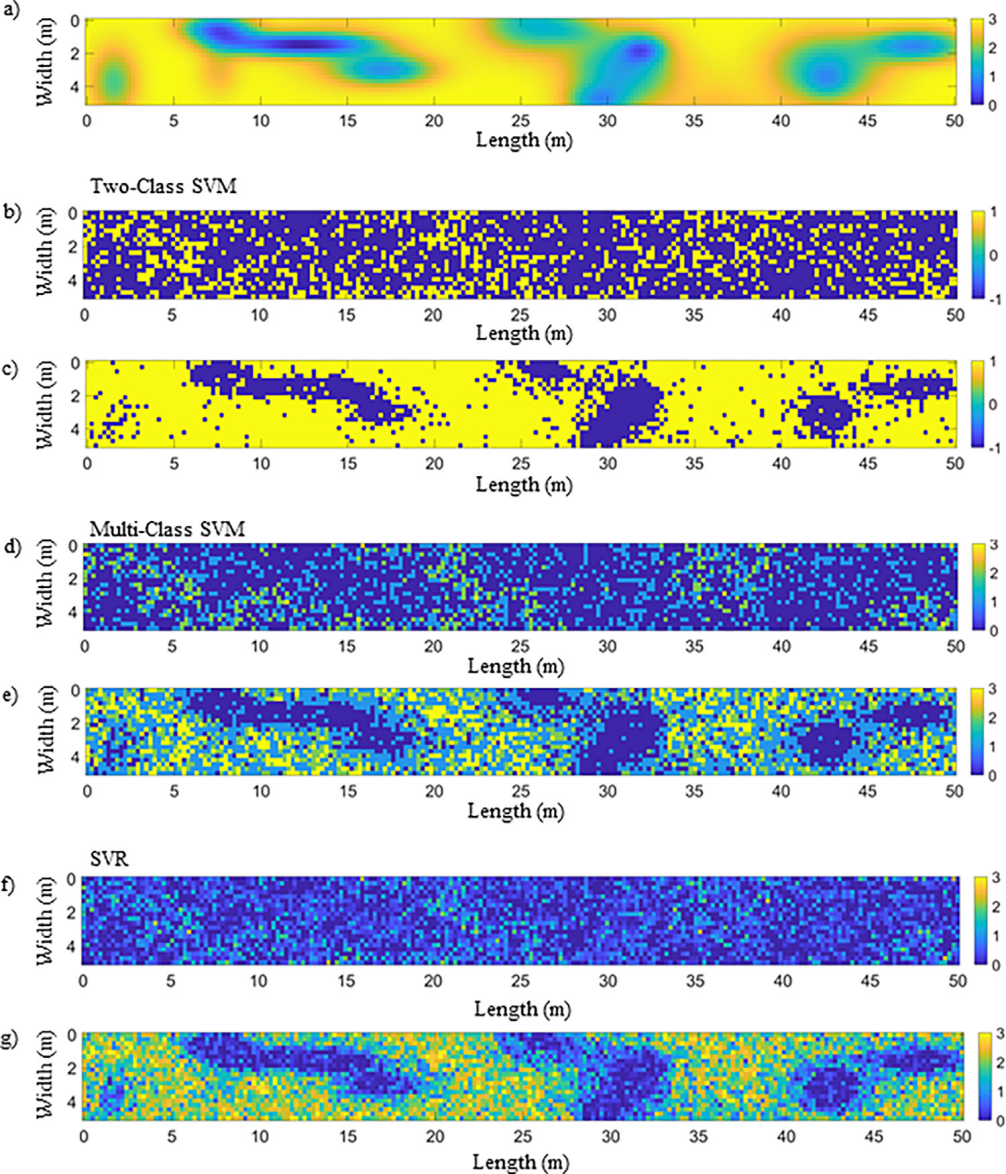
Results of inverse radar data (SNR = 20 dB); b) Two-Class SVM, global approach, without hybridization; c) Two-Class SVM with hybridization; d) Multi-Class SVM, global approach, without hybridization; e) Multi-Class SVM with hybridization; f) SVR, global approach, without hybridization; g) SVR with hybridization.

In this application, with Matlab, a white Gaussian noise to increase dispersion and evaluate robustness is introduced with SNR (Signal-Noise Ratio) = 20 dB. A windowing process is applied around the region of interest (interface), allowing less relevant parts of the signal to be discarded and consequently reducing computation time. Currently, windowing is performed manually. The authors base it on the global minimum (around 0.8 ns) of the first echo, which results from the combination of the surface echo and the direct wave. An additional 271 points (for BBTM) or 381 points (for BBSG) are added to define the window width. The window used is either the Blackman-Harris or the Tukey window. Additionally, windowing helps reduce the size of input signals by retaining only the region of interest (around the interface), thereby improving the performance of the Support Vector Machine models. Knowing the dielectric permittivity (e.g. using Full-Waveform Inversion as in Fig. 9) of the wearing course, its **observable** is appended at the end of each A-scan. The newly preprocessed dataset then serves as input to the SVM/SVR machine learning model and related Gaussian kernel method, known as the **hybrid model**. Using a Bayesian approach, the optimal values of the hyperparameters (kernel, C, γ) are determined, leading to a reduction of the objective function. By examining the resulting confusion matrices (Fig. 10) and electromagnetic maps (Fig. 11), the impact of a hybrid data processing approach on both performance metrics and cartographic representation can be observed. The full details of the methodology and choices are provided in the reference article [1].

By examining the confusion matrices corresponding to the datasets shown in Fig. 5, Fig. 6, an improvement in true positive classification can be observed. For the Two-Class SVM, 3078 A-scans were correctly classified using the hybrid model (Fig. 10a), compared to only 964 with the global approach (Fig. 10b). A similar improvement is seen with the Multi-Class SVM method, with 1748 A-scans correctly classified using the hybrid model (Fig. 10c), versus 653 with the global approach (Fig. 10d).

By comparing the different methods and approaches (Table 3), the performance improvement from the global approach to the hybrid model can be observed, thanks to the windowing technique combined with the integration of the wearing course permittivity.

**Table 3:** Performance and methods with different approach (SNR = 20 dB).

Method	Approach	Recall	Precision	F1-Score	RMSE	R²
Two-Class SVM	Global	0.52	0.77	0.62	-	-
	Hybrid	**0.88**	**0.93**	**0.91**	-	-
Multi-Class SVM	Global	0.24	0.33	0.12	-	-
	Hybrid	**0.5**	**0.5**	**0.43**	-	-
SVR	Global	-	-	-	1.93	15
	Hybrid	-	-	-	**0.8**	**72**

By mapping the output of the machine learning model for each A-scan, an electromagnetic representation of the tack coat geometry can be generated (Fig. 11). By comparing with the ground truth (Fig. 11a), the classification results for each method (Two-class SVM, Multi-Class SVM, and SVR) can be observed, providing a global approach in contrast to the hybrid model.

The methodology developed around these databases highlights the feasibility of characterizing very thin pavement layers using machine learning techniques. Furthermore, incorporating the physical parameters describing each signal as input features enables a hybrid data processing approach, enhancing algorithm performance across distinct datasets. For further details, we encourage the reader to refer to article [1].

## Limitations

This database is generated in 2 dimensions (length and depth) for reasons of computing time. The use of more powerful computing machines could enable the creation of a 3-dimensional database. The use of other antennas could also be envisaged.

## Ethics Statement

The present work did not involve the use of human subjects, animal experiments, or data collected from social media platforms.

## CRediT Author Statement

**Grégory Andreoli:** Conceptualization, Methodology, Investigation, write original draft; **Amine Ihamouten:** Conceptualization, Supervision, Writing and Review & Editing, Validation; **Xavier Dérobert:** Supervision, Review & Editing, Validation.

## Acknowledgments

This research did not receive any specific grant from funding agencies in the public, commercial, or not-for-profit sectors.

## Data Availability

DataverseSynthetic_GPR_Database.zip (Reference data).
